# Progression of liver stiffness predicts clinical events in HIV/HCV-coinfected patients with compensated cirrhosis

**DOI:** 10.1186/s12879-015-1291-3

**Published:** 2015-12-07

**Authors:** Nicolás Merchante, Francisco Téllez, Antonio Rivero-Juárez, Maria José Ríos-Villegas, Dolores Merino, Manuel Márquez-Solero, Mohamed Omar, Eva Recio, Montserrat Pérez-Pérez, Ángela Camacho, Sara Macías-Dorado, Juan Macías, Sandra Lorenzo-Moncada, Antonio Rivero, Juan A. Pineda, on behalf of the Grupo Andaluz para el Estudio de las Hepatitis Víricas (HEPAVIR) de la Sociedad Andaluza de Enfermedades Infecciosas (SAEI)

**Affiliations:** Unidad Clínica de Enfermedades Infecciosas y Microbiología. Instituto de Biomedicina de Sevilla (IBiS). Hospital Universitario de Valme, Avenida de Bellavista s/n, 41014 Sevilla, Spain; Unidad de Gestión Clínica de Enfermedades Infecciosas y Microbiología. Hospital de La Línea de la Concepción, AGS Campo de Gibraltar, Cádiz, Spain; Unidad de Enfermedades Infecciosas. Hospital Universitario Reina Sofía. Instituto Maimónides de Investigación Biomédica de Córdoba (IMIBIC), Córdoba, Spain; Unidad de Enfermedades Infecciosas. Hospital Universitario Virgen Macarena, Sevilla, Spain; Unidad de Gestión Clínica de Enfermedades Infecciosas. Complejo Hospitalario de Huelva, Huelva, Spain; Unidad de Gestión Clínica de Enfermedades Infecciosas. Hospital Virgen de la Victoria. Complejo Hospitalario de Málaga, Málaga, Spain; Unidad de Enfermedades Infecciosas. Complejo Hospitalario de Jaén, Jaén, Spain

**Keywords:** HIV/AIDS, HCV, Liver cirrhosis, Liver stiffness, Hepatocellular carcinoma

## Abstract

**Background:**

Our objective was to assess the predictive value of the changes of liver stiffness (LS) for clinical outcome in HIV/HCV-coinfected patients with compensated liver cirrhosis and a LS value < 40 kPa.

**Methods:**

Prospective cohort of 275 HIV/HCV-coinfected patients with cirrhosis, no previous liver decompensation (LD) and LS < 40 kPa. The time from diagnosis to LD and/or hepatocellular carcinoma (HCC) and the predictors of this outcome were evaluated. Significant progression of LS was defined as an increase ≥ 30 % over the baseline value at any time during the follow-up.

**Results:**

After a median (Q1-Q3) follow-up of 32 (20–48) months, 19 (6.9 %, 95 % CI: 3.8 %–9.9 %) patients developed a first LD and/or HCC. At the end of the follow-up, 247 (90 %) patients had undergone a further LS examination. Of them, 77 (31 %) patients had a significant progression of LS. The mean (SD) survival time free of LD and/or HCC was 67 (3) and 77 (1) months in patients with or without significant progression of LS (*p* = 0.01). Significant progression of LS was an independent predictor of LD and/or HCC (Adjusted Hazard Ratio 4.63; 95 % confidence interval: 1.34–16.02; *p* = 0.015).

**Conclusions:**

Significant progression of LS is associated with a higher risk of clinical events in HIV/HCV-coinfected patients with compensated cirrhosis and LS < 40 kPa.

## Background

Liver stiffness (LS), assessed by transient elastography (TE), is a surrogate marker of portal hypertension (PH) in patients with end-stage liver disease (ESLD). Thus, several studies have demonstrated that LS correlates with the hepatic venous pressure gradient, an invasive measure of the portal pressure [1–3]. Besides, the presence of oesophageal varices (EV), a major complication of PH, can be predicted by LS [4–8]. TE is painless, non-invasive and can be easily performed in outpatient setting, which has contributed to widespread use of this procedure.

In the recent years, several investigations have confirmed that LS may also provide prognostic information in patients with ESLD. Thus, a prospective cohort study conducted in 239 human immunodeficiency virus (HIV)/hepatitis C virus (HCV)-coinfected patients with a new diagnosis of cirrhosis demonstrated that LS values at diagnosis predict the clinical outcome in these individuals [9]. In that study, a LS ≥ 40 kiloPascals (kPa) at the date of the diagnosis of cirrhosis was an independent predictor of the emergence of liver decompensation (LD) in this population [9].

New direct-acting antiviral agents against HCV have notably increased the rates of sustained virological response (SVR) in HCV-infected patients, including those who are coinfected with HIV and those harbouring advanced liver disease. However, patients with established ESLD are a unique scenario where efficacy rates might be slightly lower and the clinical benefit of the achievement of SVR less marked. For these reasons, the prediction of clinical events is still critical in the management of ESLD. In this sense, some clinical decisions, as the indication of liver transplantation, are based on the risk of developing LD or death. In clinical practice, this prediction is commonly made on the basis of clinical and routine biochemical parameters, which are combined in prognostic scores as the Child-Turcotte-Pugh (CTP) [10] or the Model for the End-Stage Liver Disease (MELD) scores [11], and, less commonly, on the basis of the invasive measurement of portal pressure. As stated above, a value of LS ≥ 40 kPa at the time of diagnosis of cirrhosis adds additional prognostic information to that provided by CTP or MELD scores. However, high-risk patients, as those harbouring elavated values of LS, would probably benefit less from therapeutic and preventive measures. For these reasons, clinical investigation should focus on implementing the prediction of events in those individuals with intermediate or low risk, i.e. patients with LS values < 40 kPa, which represent three quarters of the overall HIV/HCV-coinfected cirrhotic patient population [9]. In this sense, we hypothesized that the changes of LS over time may have an impact on the clinical outcome of patients with ESLD.

The objective of our study was to assess the predictive value of the changes of LS for clinical outcome in HIV/HCV-coinfected patients with compensated liver cirrhosis and a LS value < 40 kPa.

## Patients and methods

### Study design and patients

This was an analysis of the prospective HEPAVIR cirrhosis cohort, in which patients from 7 hospitals in Andalusia, Southern Spain are recruited. This prospective cohort of HIV/HCV-coinfected patients with compensated liver cirrhosis was created in February 2006. From this date, all consecutive HIV/HCV-coinfected patients with detectable serum HCV-RNA and a new diagnosis of liver cirrhosis, based on the presence of LS > 14 kPa [12], and no evidence of toxic, metabolic or autoimmune liver disease, were enrolled, provided that they had not developed a LD before entering the cohort. For this study, those patients who had a LS < 40 kPa at the time of the diagnosis of cirrhosis were selected. Subjects who presented with a LD or hepatocellular carcinoma (HCC) at the time of cirrhosis diagnosis were excluded.

### Follow-up

Patients were evaluated at least every 6 months, including an assessment of LD and the performance of routine laboratory tests. TE examinations were performed by a single experienced operator in each center following a standardized technique [13]. After the initial LS measurement, periodic TE examinations were performed usually every 12 months, according to the caring physician criteria and the availability in each center.

The management of ESLD in the cohort was performed as previously reported [9]. LD, which included portal hypertensive gastrointestinal bleeding (PHGB), ascites, hepatorenal syndrome (HRS), spontaneous bacterial peritonitis (SBP) and hepatic encephalopathy (HE), and HCC were diagnosed and managed according to criteria stated elsewhere [14–16]. Therapy against HCV was offered during follow-up according to the physician criteria, usually following guidelines in force during the follow-up or expert opinions [17].

Patients were prospectively seen until death, liver transplant or the censoring date (30 November 2012). Vital status and causes of death were recorded according to clinical records. Patients lost to the follow-up or their next of kin were contacted via telephone whenever possible.

### Statistical analysis

Comparisons of continuous variables were made using the Student *t* test or the Mann–Whitney *U* test, depending on the normality of distributions. The Wilcoxon test was used to compare values of continuous variables at different time-points. The chi-square and the Fisher tests were used for comparisons of categorical variables.

The primary end-point of the study was the emergence of a first episode of LD and/or HCC. The relationship between the time to the emergence of the primary end-point and variables that could potentially be associated, which included baseline LS and changes of LS during follow-up, were analysed.

For survival analyses, we considered the baseline time-point the date of the first TE examination in which the diagnosis of cirrhosis was established. The time-to-event was computed as the months elapsed from baseline to the emergence of the primary outcome. Kaplan-Meier estimates of the cumulative probability of survival were compared using the log-tank test. For these analyses continuous variables were categorized according to the median value or cut-off points considered to be clinically relevant. Baseline LS was also categorized by clinically relevant cut-off points based on previous studies. The definition of significant progression was developed after the performance of exploratory analyses. Thus, different analytic approaches were explored and that with the better performance was chosen. Consequently, significant progression of LS was defined as an increment of LS at any time during the follow-up ≥ 30 % over the baseline LS. According to this, patients were divided in progressors, those who showed a significant progression of LS, and non-progressors, the rest of the population. Those variables with a *p* value ≤ 0.1 on the univariate analysis were entered in time-dependent Cox proportional hazard models, which also included age, sex and the achievement of SVR during follow-up. Finally, the presence of statistical interactions were evaluated by means of multivariate Cox regression analyses. Associations with a *p* <0.05 were considered significant in the multivariate analysis. The hazard ratio (HR) and the respective 95 % confidence interval (CI) were calculated. Comparisons between the areas under the receptor operating characteristic (AUROC) curves were performed using the Hanley and McNeil test. The statistical analysis was carried out using the SPSS 22 Statistical Software Package (SPSS; Chicago, Illinois, USA) and the STATA version 9 (Stata Corp LP, College Station, TX, USA).

### Ethical aspects

The study was designed and conducted following the Helsinki declaration. The Ethics committee of the Hospital Universitario de Valme approved the study. All the participant subjects gave their written consent to participate in the study.

## Results

### Patient features

Three hundred and forty-nine HIV/HCV-coinfected patients with a diagnosis of cirrhosis on the basis of a LS ≥ 14 kPa and who fulfilled inclusion criteria had been recruited in the HEPAVIR cohort until November 2012. Of them, 275 (79 %) patients had a baseline LS < 40 kPa and were selected for this study. The main characteristics of the study population are summarized in Table 1. During follow-up, 107 (39 %) patients started therapy against HCV. At the end of study period, 13 out of these 107 patients were still receiving such a therapy. SVR was achieved in 29 (31 %) of the remaining 94 patients.

**Table 1:** Characteristics of the study population (*n* = 275)

Parameter	Value
Age (years)^a^	44 (42–48)
Male gender, no. (%)	245 (89)
Daily alcohol intake > 50 grs/day, no. (%)	40 (14)
Previous intravenous drug users, no. (%)	250 (91)
Previous therapy against HCV, no (%)	57 (21)
HCV genotype, no. (%)^b^	
1	165 (61)
2	2 (1)
3	61 (23)
4	41 (15)
HCV RNA load (log_10_ IU/mL)^a,c^	6.17 (5.68–6.69)
Positive hepatitis B virus surface antigen, no (%)	8 (3)
Serum alanine aminotransferase (IU/L)^a^	74 (48–105)
Serum aspartate aminotransferase (IU/L)^a^	72 (47–104)
Total bilirrubin (mg/dL)^a^	0.7 (0.5–1.02)
Platelet count (/mm^3^)^a^	130000 (94000–171750)
HIV RNA load < 50 copies/mL, no. (%)	184 (67)
CD4 cells/mL^a^	459 (265–626)
ART^d^, no (%)	256 (83)
MELD^e^ score^a,f^	7 (6–9)
Child-Turcotte-Pugh class, no (%)	
A	248 (90)
B	27 (10)
Liver stiffness (kPa)^a^	20 (17–27)

^a^Median (Q1-Q3)

Available in ^b^269 and

^c^254 patients

^d^ART: Antiretroviral therapy

^e^MELD: Model for end-stage liver disease

^f^Available in 251 patients

### LD and mortality

The median (Q1-Q3) follow-up was 32 (20–48) months. Fourteen (5 %) patients were lost to follow-up. Nineteen (6.9 %, 95 % CI: 3.8 %–9.9 %) patients developed a first LD and/or HCC as the first cirrhosis complication during the follow-up. The incidence rate of LD and/or HCC was 4.6 per 100 person-years (95 % CI: 2.9 %–7.3 %). Ascites was the most common type of first LD, appearing in 10 (53 %) patients. PHGB occurred in 2 (10 %) patients and SBP in 1 (5 %). HCC was the first hepatic event in 6 (32 %) patients. The median (Q1-Q3) time to the development of a LD and/or HCC was 17 (11–28) months.

Twenty-two (7.6 %, 95 % CI: 4.4 %–10.7 %) patients died during follow-up. The mortality rate was 4.1 deaths per 100 person-years (95 % CI: 2.6–6.1). In 12 patients, death was related with liver failure. Thus, the liver-related mortality rate was 3.2 deaths per 100 person-years (95 % CI: 1.8–5.6). Specific liver-related causes of death were: 3 patients died due to HE, 2 due to PHGB, 1 due to HRS, 1 due to SBP and 5 due HCC. Ten patients died because of non-liver related causes: 3 patients due to cerebral bleeding, 2 patients as a result of a non-AIDS related neoplasm, 2 patients due to bacteriemic pneumococcal pneumonia, 1 patient owing to *Pneumocystis jirovecii* pneumonia, 1 patient due to acute renal failure other than HRS and 1 patient committed suicide. Of these 10 patients who died because of non-liver related causes, only 1 had previously developed a LD. No patient underwent a liver transplant.

### Association between baseline LS and the emergence of hepatic events

**Fig. 1:**
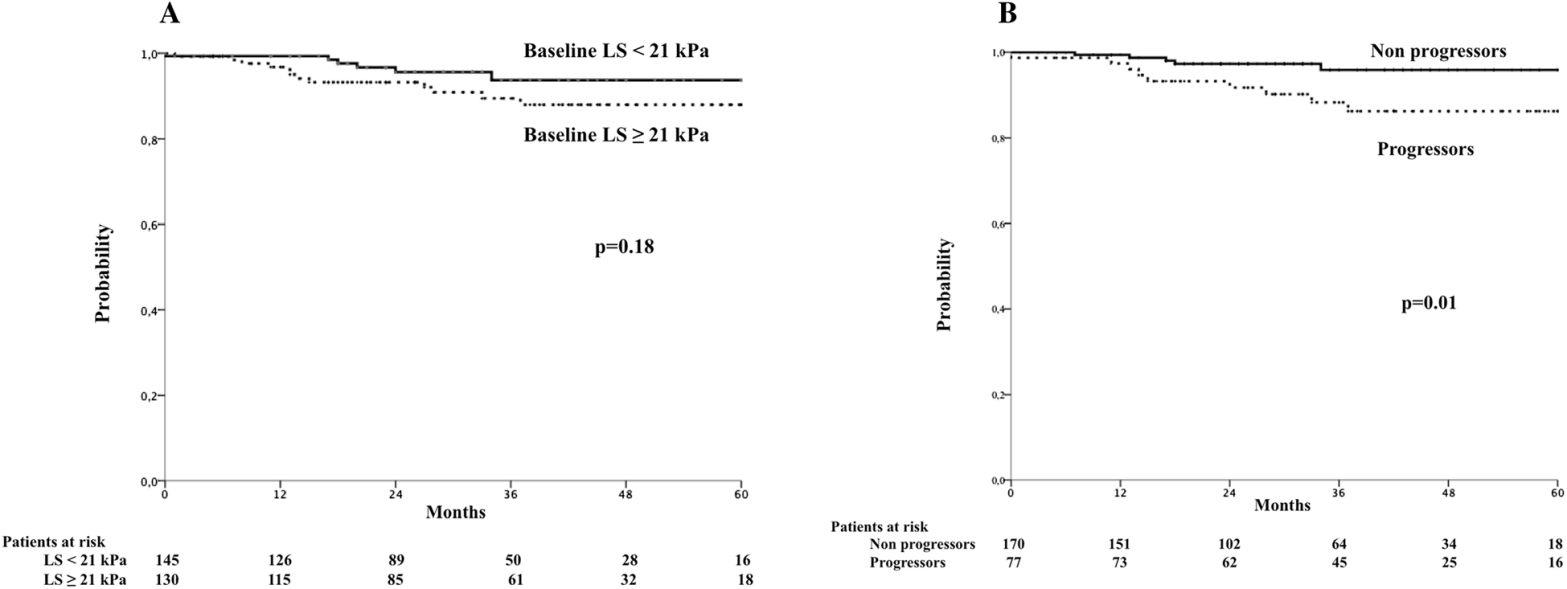
Liver stiffness and probability of liver-related events during follow-up. **a** Probability of remaining free of developing a hepatic decompensation and/or hepatocellular carcinoma according to baseline liver stiffness. LS, liver stiffness. kPa, KiloPascals. **b** Probability of remaining free of developing a hepatic decompensation and/or hepatocellular carcinoma according to the evolution of liver stiffness during the follow-up. Progressors are defined as those patients showing an increase of LS ≥ 30 % over the baseline value at any time during the follow-up

Higher baseline LS values tended to be associated with developing a first LD and/or HCC (Table 2 and Fig. 1a). The probabilities of remaining free of LD and/or HCC at 1 year, 2 years, 3 years and 4 years were 99, 97, 94 and 94 %, respectively, for patients with a baseline LS < 21 kPa whereas they were 97, 93, 89 and 87 % for patients with a baseline LS ≥ 21 kPa. Comparisons between groups using different cut-off points of baseline LS did not yield statistically significant results. Thus, the respective figures for patients with baseline LS < 30 kPa were 99, 95, 91 and 91 %, whereas for those with a baseline LS ≥ 30 kPa were 96, 96, 93 and 89 %, respectively.

**Table 2:** Univariate associations between emergence of and episode of hepatic decompensation and/or HCC and other parameters (*n* = 275)

Parameter	Category	No. (%) with decompensation	*P* Bivariate (Log rank test)
Sex	Male	16 (6)	
	Female	3 (10)	0.4
Age	< 44 years	6 (5)	
	≥ 44 years	13 (8)	0.2
Hepatitis B virus surface antigen	Negative	17 (6)	
	Positive	2 (25)	0.15
HCV genotype	1–4	13 (6)	
	2–3	6 (9)	0.4
HCV RNA load (log_10_ IU/mL)^a^	< 6.7	13 (7)	
	≥ 6.7	5 (8)	0.5
Therapy against HCV during follow-up	No therapy or therapy without SVR^b^	16 (6)	
	SVR^b^	3 (11)	0.6
CD4 cell count (cells/mm^3^)	< 200	5 (8)	
	≥ 200	14 (6)	0.5
Platelet count (/mm^3^)	< 150.000	16 (9)	
	≥ 150.000	3 (3)	0.061
APRI^c^ score	< 1.5	9 (7)	
	≥ 1.5	10 (7)	0.9
Child-Turcotte-Pugh class	A	15 (6)	
	B	4 (15)	0.002
MELD^d^ score^e^	< 14	17 (7)	
	≥ 14	0 (0)	0.6
Liver stiffness (kPa)	< 21	7 (5)	
	≥ 21	12 (9)	0.18
Progression of liver stiffness ≥ 30 % from baseline^f^	No	5 (3)	
	Yes	10 (13)	0.011

^a^Available in 254 patients

^b^SVR: Sustained virological response

^c^APRI: AST platelet ratio index

^d^Model for end-stage liver disease

^e^Available in 251 patients

^f^Evaluable in 247 patients

### Changes of LS during follow-up and association with the emergence of hepatic events

**Fig. 2:**
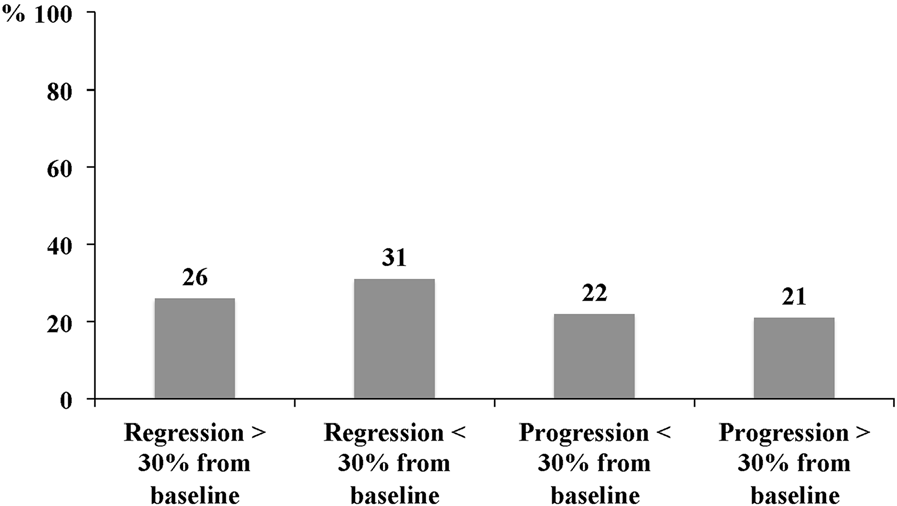
Distribution of patients according to the evolution of liver stiffness from baseline to the end of follow-up in the 247 patients who underwent a liver stiffness examination at the end of the follow-up

At the end of the follow-up, 247 (90 %) patients had undergone at least one LS examination after that carried out at screening for entering the cohort. Twelve (4 %) patients were included in the cohort in the last 12 months before the censoring date and had only a LS determination at baseline. Two patients (1 %) were lost to the follow-up before a second examination could be performed. In the remaining 14 patients (5 %), a second LS examination was not available. In 120 (44 %) patients, 4 or more LS examinations were performed. One hundred and forty (57 %) patients underwent LS examinations every 6 months. The median (Q1-Q3) LS at baseline and at the end of the follow-up was 20 (17–27) kPa and 19 (13–28) kPa, respectively (*p* = 0.3). Figure 2 shows the distribution of patients in different groups according to the evolution of LS from baseline to the end of follow-up in the 247 patients who underwent a LS examination at the end of the follow-up. LS increased from baseline to the end of the study period in 107 (43 %) patients.

Seventy-seven (31 %) patients showed an increase of LS ≥ 30 % from baseline at any time during the follow-up. The occurrence of progression ≥ 30 % of LS at any time during the follow-up was associated with the emergence of a LD and/or HCC (Table 2 and Fig. 1b). The mean (standard deviation [SD]) survival time free of LD and/or HCC was 67 (3) and 77 (1) months in those patients with or without an increase ≥ 30 % in LS during the follow-up, respectively (*p* = 0.01). The probabilities of remaining free of LD and/or HCC at 1 year, 2 years, 3 years and 4 years were 99, 97, 96 and 96 %, respectively, for patients without a significant progression of LS during the follow-up whereas they were 97, 93, 88 and 86 % for patients in whom LS increased ≥ 30 % from baseline at any time during the follow-up. Significant progression of LS occurred before the emergence of LD and/or HCC in the 10 patients who developed this event during follow-up and had a significant progression of LS. Thus, the median (Q1-Q3) elapsed time from LS progression to LD was 6 (4–13) months.

### Independent predictors of the emergence of LD and/or HCC

In order to compare the predictive value of baseline LS and progression of LS for clinical outcomes, two multivariate models were generated: one included baseline LS and another progression of LS during the follow-up. For the latter, we previously explored if progression of LS covariated with time in a time-dependent Cox model, and we were able to rule it out. Table 3 summarizes these analyses. In the multivariate model that included baseline LS, age, sex, previous therapy against HCV and CTP stage were independently associated with developing a LD and/or HCC (Table 3). Baseline LS was not independently associated with the primary end-point. In the multivariate model that included progression of LS, the independent predictors of developing a LD and/or HCC were age, previous therapy against HCV, consecution of SVR during follow-up, CTP stage and progression of LS ≥ 30 % at any time during the follow-up (Table 3).

**Table 3:** Multivariate models for the prediction of hepatic decompensation and/or HCC

	Parameter	Multivariate hazard ratio (95 % CI)	*P* multivariate
Model 1			
	Age (years)^a^	1.11 (1.01–1.16)	0.028
	Female sex	3.2 (0.59–17.16)	0.177
	SVR^b^ during follow-up	0.46 (0.12–1.81)	0.266
	Platelet count (/mm^3^) ≥ 150.000	4.01 (0.81–19.85)	0.089
	Child-Turcotte-Pugh class A	1.49 (0.18–12.41)	0.713
	Baseline liver stiffness ≥ 21 kPa	1.9 (0.59–6.07)	0.279
Model 2			
	Age (years)^a^	1.06 (0.99–1.13)	0.053
	Female sex	2.36 (0.46–12.09)	0.302
	SVR^b^ during follow-up	0.26 (0.06–1.14)	0.075
	Platelet count (/mm^3^) ≥ 150.000	2.99 (0.61–14.77)	0.178
	Child-Turcotte-Pugh class A	1.88 (0.23–15.55)	0.556
	Progression of liver stiffness ≥ 30 % from baseline	4.63 (1.34–16.02)	0.015

^a^Considered as a continuous variable for multivariate analyses

^b^SVR: Sustained virological response. Model 1 indicates the multivariate model based on baseline liver stiffness. Model 2 indicates the multivariate model based on the progression of liver stiffness

Additionally, we compared the ability of baseline LS and progression of LS during follow-up to predict the primary outcome. Thus, we computed the respective AUROC of the multivariate models including either baseline LS or progression of LS, obtaining a higher AUROC for the progression of LS-based model. Namely, the AUROC (95 % CI) for baseline LS including multivariate model was 0.609 (0.471–0.748), while the respective figure for progression of LS including multivariate model was 0.680 (0.541–0.818) (*p* = 0.1).

## Discussion

This study demonstrates that progression of LS is associated with the emergence of LD and/or HCC in HIV/HCV-coinfected patients with ESLD and LS < 40 kPa. Although the risk of LD was globally low in the short and mid-term in this population, patients with a significant progression of LS during follow-up, as defined in this study, had a higher risk of clinical events in the years after the diagnosis of cirrhosis. Besides, this association was independent of other prognostic factors as CTP score in multivariate analyses. Finally, changes in LS during the follow-up have a stronger impact on the outcome than the initial value of LS, suggesting that prediction of events should be based on changes in LS rather than on a single value in patients with a LS < 40 kPa.

It has been demonstrated that LS values at diagnosis of cirrhosis predict clinical outcome in patients with ESLD in different settings [9, 18–23]. However, the clinical meaning of LS changes and its influence in the prognosis of cirrhotic patients is less well-established. In a French study [24], which included 150 patients with primary biliary cirrhosis who underwent at least 2 TE examinations, LS progression was associated with a composite end-point including LD, liver transplant and death of any cause. However, this study included patients with different stages of liver fibrosis, with only 8 % of them having cirrhosis according to baseline LS values. Un updated analysis of the previous cohort showing similar results has been very recently reported [25]. A second study [22], conducted in 103 Asian patients with hepatitis B virus chronic infection undergoing antiviral therapy with F3 or F4 fibrosis, has reported that early changes in LS, defined as those occurring in the first 6 months of follow-up, were associated with a composite end-point of LD, HCC and liver-related death.

In our opinion, the present study has several methodological advantages that solve some of the questions raised by the above-mentioned studies [22, 24, 25]. First, it includes patients with cirrhosis exclusively, all of them diagnosed by the same method, TE. Second, we have excluded those patients harbouring a LS ≥ 40 kPa, in order to avoid potential confusions due to the strong impact that high baseline LS can exert on the outcome. Third, we have performed comparisons between the prognostic value of baseline LS and the changes of LS that suggest a superior performance of the latter. Finally, the criteria used in this study for defining significant progression of LS was chosen after performing different exploratory sensitivity analyses looking for that with the best performance, as there are not enough studies so far that have established a “consensus definition”. After that, we classified patients according to the occurrence of an increase ≥ 30 % over the baseline value at any time during the follow-up for several reasons. First, it is a high cut-off value, which excludes potential changes of LS due to normal variability in measures between examinations. Second, it takes into account the initial value of LS, which modulates the impact of the changes by considering also the risk level from what the patient starts. Third, it was the analytic approach that performed the best.

Our results contribute to the growing evidence that favours the routine clinical application of LS measurement in the management of patients with ESLD. Studies have shown that mild PH can be predicted based on routine clinical data and the evaluation of LS [26, 27]. Besides, the presence of EV requiring therapy can be excluded on the basis of the assessment of LS [4–8]. Notably, the risk of HCC development varies according to the pattern of changes in LS values and serial measurements of LS have been proposed as a monitoring tool for estimating the risk of HCC development in patients chronically infected by hepatitis B virus [28]. Finally, PH-related complications can be predicted by TE. LS values at the diagnosis of cirrhosis predict clinical outcome and identify a group of patients at a high risk for decompensation in the short term, those harbouring a LS ≥ 40 kPa [9]. As shown in this study, significant progression of LS may also help to detect early an increased risk for LD and/or HCC in otherwise asymptomatic individuals with a LS < 40 kPa.

The prediction of clinical events is of a paramount importance in the management of patients with ESLD. Thus, some decisions, as the indication of liver transplantation, are based on the risk of developing LD or death. Besides, the allocation of candidates for transplantation in the waiting list in most liver transplant programs is based on classical prognostic scores as the MELD score [11]. However, overall prognosis is very poor in patients with decompensated cirrhosis in certain populations, as HIV/HCV-coinfected patients [29]. For this reason, an accurate prediction of complications should be made early in the course of ESLD in order to optimize measures. In this sense, we have previously demonstrated that a LS ≥ 40 kPa provides additional prognostic information to that given by the CTP or the MELD score in HIV/HCV-coinfected patients with compensated cirrhosis [9]. The present study confirms that LS also adds prognostic information to that provided by classical scores in patients with LS < 40 kPa. To note, LS changes predict more accurately the risk of hepatic events than baseline LS and anticipate the development of clinical events. In fact, LS progression occurred several months before the emergence LD in our study. Consequently, sequential measurements of LS may be considered as a strategy to monitor the risk of events in this population, as it has also been proposed by others [30].

Our study may have some limitations. First, LS measurements during follow-up were performed according to the caring physician criteria and the availability at each hospital. However, the great majority of patients were monitored at a minimum every 12 months and more than one half of the patients were monitored every 6 months. We cannot completely exclude that more frequent LS monitoring would have identified changes that may have been not detected with this interval, although this seems very unlikely. Besides, the interval between examinations was quite similar in another studies assessing this issue [24, 25]. Second, the number of events was relatively low and the follow-up period somewhat short for a low-risk population. This might have precluded us to identify some independent associations in multivariate analysis and might have influenced the performance of the multivariate models. However, even with this potential limitation, this study has been able to prove that the progression of LS is an independent predictor of clinical events and that adds prognostic information beyond that provided by the baseline LS value and CTP score. On the other hand, we present data from a large, prospective cohort of HIV/HCV-coinfected patients with compensated cirrhosis diagnosed using the same method. Besides, we used a definition of progression of LS that was strict and easy to apply in the routine daily practise, while the primary end-point in our study was the emergence of LD and/or HCC, which we assessed after a median follow-up of 2.6 years. These are strengths of our study.

## Conclusions

In summary, significant progression of LS is associated with a higher risk of clinical events in HIV/HCV-coinfected patients with compensated cirrhosis and a LS < 40 kPa. Besides, changes in LS provide additional prognostic information to that given by classical prognostic scores, as CTP or MELD scores. Finally, in this overall low-risk population, the prediction of clinical events should be based ideally on the changes of LS better that on a single LS measurement. Thus, sequential monitoring of LS in compensated cirrhotic patients is desirable in order to properly assess the prognosis.

## Abbreviations

LS: Liver stiffness
TE: Transient elastography
PH: Portal hypertension
ESLD: End-stage liver disease
EV: Esophagueal varices
HIV: Human immunodeficiency virus
HCV: Hepatitis C virus
kPA: KiloPascals
LD: Liver decompensation
SVR: Sustained virological response
CTP: Child-Turcotte-Pugh
MELD: Model for end-stage liver disease
HCC: Hepatocellular carcinoma
PHGB: Portal hypertensive gastrointestinal bleeding
HRS: Hepatorenal syndrome
SBP: Spontaneous bacterial peritonitis
HE: Hepatic encephalopathy
HR: Hazard ratio
CI: Confidence interval
AUROC: Area under the receptor operating characteristic
